# Meddlesome Midwifery

**Published:** 1889-03

**Authors:** Samuel L. Eaton

**Affiliations:** Watkins, N. Y.


### MEDDLESOME MIDWIFERY.

SAMUEL L. EATON, M. D., WATKINS, N. Y.

This term indicates a fault for which the laity is perhaps fully as much to blame as is the profession. I have often been surprised to observe the child-like glee with which people of years and discretion welcome the chance to "do something" for their sick friends, even when they are conscious that what they are doing is useless or worse than useless. The physician instinctively recognizes this craving for action ; and, yielding to it, is led into various kinds of meddling—obstetric and otherwise. It is the former variety that I wish to illustrate by a brief history of two of my patients.

Mrs. J., aged twenty-seven, mother of two healthy children of three and five years respectively, sent for me one morning last June. She had just been delivered of a three months' fœtus, and was very anxious about the after-birth, which, she informed me, had always been a source of trouble on similar occasions in the past. At one time the placenta had been forcibly removed by difficult and painful measures; at another time it had been allowed to remain, with the result of poisoning the patient by septic absorption. I found her flowing quite freely but not alarmingly, the mouth of the womb closed, the patient nervous. I prescribed Sabina, to be taken at half-hour intervals, and told her husband that I expected the placenta would come all right. He was quite incredulous, but allowed me to depart with the promise of returning in two hours. At the expiration of that time I found that the placenta had just come away, and the patient was comfortable. Nothing hindered her making a speedy recovery.

A single miscarriage ought to be enough for any one year, but in December the same accident happened to my patient. The fœtus was born as before, without much trouble, at about the third month. The placenta did not make its appearance, and the flow was very slight. I prescribed Belladonna on account of a throbbing headache, and went my way—this time without meeting with any opposition. On the following day the patient presented no headache, no hemorrhage, no symptoms of any kind. No medicine. On the third day I saw her about noon, and she was still perfectly comfortable. Prescribed Pulsatilla, to be taken every hour. Early on the morning of the fourth day I was sent for on account of a quite active hemorrhage. Found the placenta presenting at the os uteri and easily removed it with my finger. The patient again made a very rapid recovery, and has remained well up to the present writing.

The other case which I wish to cite was diametrically opposite in its important particulars. Mrs. X., aged twenty-nine, of nervo-bilious temperament and delicate constitution, approached her confinement last fall under circumstances which caused me some anxiety. In 1884 she had given birth to her first child, had gotten up too quickly, and about six weeks after its birth had suffered from alarming hemorrhages. This was under old-school treatment. In the following year she had a miscarriage at the fourth month, followed by a tedious illness, with a long-continued flow, which, taken with her previous history, seemed to indicate a real hemorrhagic diathesis. In 1886 she was delivered of a healthy boy, and at that time did fairly well. In 1887, she was again pregnant and went to term, with a naturally delicate constitution much debilitated by the heavy drafts paid since her marriage. Labor was not specially rapid, but was remarkably easy. The pains were few and far between, but, owing to a roomy pelvis and the extremely lax condition of all the fibres, very little effort was required to bring the child into the world. A few minutes after its birth I endeavored to remove the placenta by combined traction and expression, without success. I then gave a dose of Pulsatilla, and, after ten minutes, made another unsuccessful effort. I then sat down to wait, but after waiting about twenty minutes, alarmed by the extreme pallor of my patient, I investigated and found her lying in a pool, I may say a lake, of blood. Directing the nurse to administer Chloroform, I forced my hand through the nearly closed os uteri, reached the placenta, which was still partially adherent, peeled it off the side of the womb, passing my hand up to the fundus, and extracted it. The doctor was nearly as much exhausted as the patient. But the result was favorable. The sluggish womb contracted under this severe stimulus, and the hemorrhage was no longer troublesome. For two days my patient was considerably troubled with after-pains, but she made, on the whole, a good recovery.

Was this "meddlesome midwifery?" To be sure. It was a case when active meddling was necessary. A few minutes more and she would have been beyond the reach of all meddlers. I have placed these two cases in juxtaposition because they illustrate the necessity of adopting different methods to different cases. In the first case, a *laissez faire* policy, combined with prescribing the remedies which seemed to me adapted to the condition, was attended with the happiest results. In the last case, active mechanical measures were necessary to save the patient. I am quite sure that she might have been saved by purely medicinal measures. But the knowledge of the materia medica which most of us possess is, unfortunately, finite, and the exigency was too instant and pressing to admit of consulting a repertory.—*Bureau of Obstetrics, I. H. A.*

